# Phenylalanine Butyramide: A Butyrate Derivative as a Novel Inhibitor of Tyrosinase

**DOI:** 10.3390/ijms25137310

**Published:** 2024-07-03

**Authors:** Ritamaria Di Lorenzo, Vincenzo Di Lorenzo, Teresa Di Serio, Adua Marzocchi, Lucia Ricci, Eleonora Vardaro, Giovanni Greco, Maria Maisto, Lucia Grumetto, Vincenzo Piccolo, Elena Morelli, Sonia Laneri

**Affiliations:** 1 Department of Pharmacy, Università degli Studi di Napoli Federico II, 80131 Naples, Italy; ritamaria.dilorenzo@unina.it (R.D.L.); ggreco@unina.it (G.G.); maria.maisto@unina.it (M.M.);; 2 Department of Organic Chemistry and Technology, Faculty of Chemical Technology and Biotechnology, Budapest University of Technology and Economics, Müegyetem rkp. 3, H-1111 Budapest, Hungary; dilorenzo.vincenzo@ttk.hu

**Keywords:** PBA, phenylalanine butyramide, hyperpigmentation, depigmentation, anti-aging, short-chain fatty acids, tyrosinase

## Abstract

Metabolites resulting from the bacterial fermentation of dietary fibers, such as short-chain fatty acids, especially butyrate, play important roles in maintaining gut health and regulating various biological effects in the skin. However, butyrate is underutilized due to its unpleasant odor. To circumvent this organoleptic unfavorable property, phenylalanine butyramide (PBA), a butyrate precursor, has been synthesized and is currently available on the market. We evaluated the inhibition of mushroom tyrosinase by butyrate and PBA through in vitro assays, finding IC_50_ values of 34.7 mM and 120.3 mM, respectively. Docking calculations using a homology model of human tyrosinase identified a putative binding mode of PBA into the catalytic site. The anti-aging and anti-spot efficacy of topical PBA was evaluated in a randomized, double-blind, parallel-arm, placebo-controlled clinical trial involving 43 women affected by photo-damage. The results of this study showed that PBA significantly improved skin conditions compared to the placebo and was well tolerated. Specifically, PBA demonstrated strong skin depigmenting activity on both UV and brown spots (UV: −12.7% and −9.9%, Bs: −20.8% and −17.7% after 15 and 30 days, respectively, *p* < 0.001). Moreover, PBA brightened and lightened the skin (ITA°: +12% and 13% after 15 and 30 days, respectively, *p* < 0.001). Finally, PBA significantly improved skin elasticity (Ua/Uf: +12.4% and +32.3% after 15 and 30 days, respectively, *p* < 0.001) and firmness (Uf: −3.2% and −14.9% after 15 and 30 days, respectively, *p* < 0.01).

## 1. Introduction

The skin is the largest organ of the human body, with various functions as a physical and immunological barrier protecting the body against environmental factors [1]. Other important functions include thermoregulation, sensoriality, and support for vitamin D synthesis [2]. 

The skin further provides a habitat for the resident microbiota [3], which under physiological conditions protects the skin from pathogenic organisms [4]. Soon after birth, bacteria start to colonize the skin and other body sites [5], and despite wide environmental variations, the skin is proficient in maintaining a stable microbial ecosystem [6]. The human microbiota lives in symbiosis with humans and actively contributes to guaranteeing skin homeostasis [7]. The skin microbiome is crucial to maintaining skin health and well-being [8]. Emerging data shows that the microbiome is a key regulator of the immune system due to its ability to maintain communication between tissues and organs [9]. Moreover, dysbiosis in the skin and/or gut microbiome is associated with an altered immune response [10]. 

Certain bacterial species have been demonstrated to modulate both pro-inflammatory and anti-inflammatory responses in the skin. The interaction between the microbiome and skin inflammation is linked to conditions like atopic dermatitis, psoriasis, connective tissue diseases, and other autoimmune inflammatory disorders such as lupus erythematosus [11]. Particularly, the impact of bacterial metabolites on immune regulation and its potential application to skin inflammation is increasingly being explored. In this regard, it has been reported that commensal microbes affect the mucosal immune system by influencing T-cell differentiation [12,13]. This pathway is mediated by short-chain fatty acids (SCFAs), which are the main group of microbiota metabolites together with tryptophan metabolites and amine derivatives including trimethylamine N-oxide [11]. SCFAs include acetate, propionate, and butyrate [14,15]. Butyrate promotes epithelial skin barrier function [16], stimulates the synthesis of hyaluronic acid in fibroblasts [17], and induces the synthesis of collagen through different metabolic processes by increasing the expression of IGF-IR [18] and reducing the activity of protein kinase MEK 1/2 [19]. Furthermore, butyrate stimulates prolidase, a dimeric enzyme involved in the final stage of protein catabolism [20], which is essential for re-using the aminoacid proline in collagen neo-synthesis and cellular growth. 

SCFAs are agonists of peroxisome proliferator-activated receptors (PPARs) [21], which are nuclear hormone receptors and comprise three different isoforms, namely PPAR-α, PPAR-γ, and PPAR-β/δ [22]. PPARs modulate a variety of skin functions, including keratinocyte proliferation, epidermal barrier formation, wound healing, melanocyte proliferation, and sebum production [23,24]. PPAR agonists have been claimed as potential agents to treat skin conditions, like malignant melanoma and melasma, by virtue of their antiproliferative activity on melanocytes and their ability to inhibit melanogenesis [25]. Recent studies have revealed that agonists of PPARα, like butyrate, may exert lightening and anti-spot action when applied topically [26]. Furthermore, PPAR-γ agonists have been found to be effective as depigmenting agents in cosmetics [27]. The binding to PPARγ decreases tyrosinase expression, thus leading to reduced melanogenesis [28].

Many treatment approaches to reduce hyperpigmentation operate by inhibiting the conversion of tyrosine to melanin, thereby acting as inhibitors of tyrosinase, the crucial regulatory enzyme for melanin biosynthesis. To our knowledge, direct tyrosinase inhibition by postbiotic metabolites, such as butyrate, has not yet been established. 

**Figure 1:**
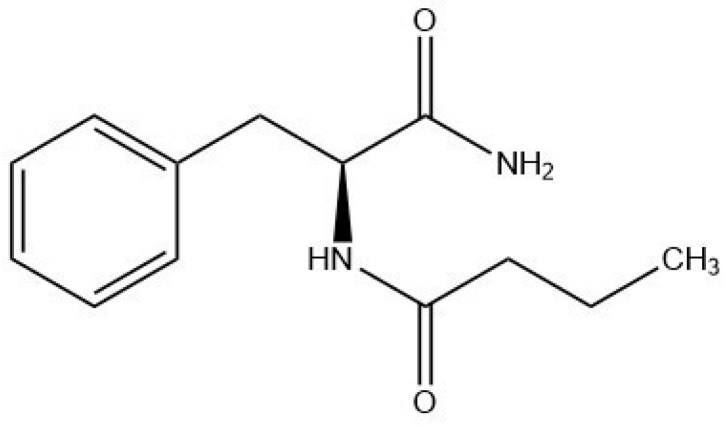
Chemical structure of phenylalanine butyramide (PBA).

Although butyrate-based products are available on the market, their adoption by consumers is still limited because of the unpleasant rancid smell of butyrate. These limitations necessitate the development of new strategies that can control the volatile nature of butyrate and mask its odor. Phenylalanine butyramide (PBA) corresponding to *N*α-butyryl-L-phenylalaninamide or *N*-1-carbamoyl-2(*S*)-phenylethyl butyramide (Figure 1), is a synthetic butyrate-releasing odorless compound that has shown valuable soothing properties on the skin [29]. PBA represents a safe alternative to butyrate because it does not exhibit genotoxicity or mutagenic activity [30] and has been described as a butyrate releaser [29,31]. 

In the present article we report, that butyrate and PBA are direct inhibitors of mushroom tyrosinase according to the results of enzymatic assays. In-silico docking simulations identified a putative binding mode for PBA in the catalytic site of the human tyrosinase isoform. More importantly, the depigmenting and lightening activity of topical PBA (30 days treatment) was evaluated in a randomized, double-blind, placebo-controlled, 2-parallel arm clinical study conducted on 43 women with hyperpigmentation or melasma. 

## 2. Results

### 2.1. Tyrosinase Inhibition

**Figure 2:**
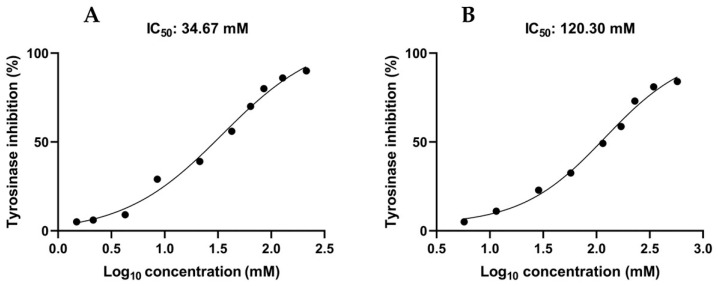
Inhibition of mushroom tyrosinase by PBA (**A**) and butyrate (**B**) calculated as IC_50_. Values represent the mean of three replicates.

Tyrosinase plays a key role in human skin pigmentation, making its inhibition a potential treatment for hyperpigmentation disorders. To assess the potential of PBA as a whitening ingredient, its ability to inhibit tyrosinase activity was evaluated through in vitro experiments using mushroom (*Agaricus bisporus*) tyrosinase, which is widely used to search for inhibitors of tyrosinase. As reported in Figure 2A, PBA showed an appreciable capability to inhibit tyrosinase in a concentration-dependent manner, with an IC_50_ of 34.7 mM. To investigate the role of individual PBA components (butyrate and L-phenylalanine) as tyrosinase inhibitors, they were tested separately. L-Phe did not exhibit any inhibitory activity, whereas butyrate inhibited tyrosinase with an IC_50_ of 120.3 mM (Figure 2B). 

### 2.2. Molecular Docking 

**Figure 3:**
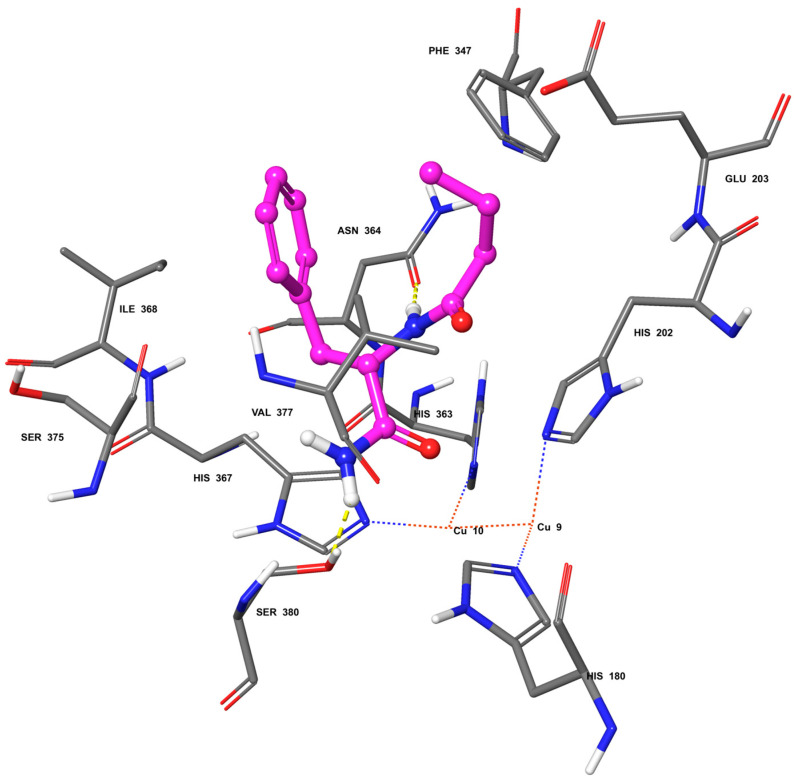
Docking model of PBA into a homology model of the human tyrosinase active site.

In silico studies were carried out using a homology model of human tyrosinase [32] leading to a putative binding mode for PBA into the catalytic site of this enzyme (Figure 3). The main intermolecular interactions established in this docking model are a H-Bond between the αNH of PBA and the side chain carbonyl oxygen of Asn364, and a H-bond between the NH_2_ of the ligand and the hydroxyl oxygen of Ser380. Hydrophobic interactions involve the phenyl moiety of PBA facing the side chain of Ile368 and the *n*-propyl chain of PBA facing the side chains of Val377 and Phe347. These results suggest that PBA may behave as an inhibitor not only of mushroom tyrosinase but also of human tyrosinase.

### 2.3. Skin Complexion and Luminosity

The individual typology angle (ITA°) is a recent objective classification system developed to assess skin pigmentation. ITA° measures constitutive pigmentation using a colorimetry measurement, and is calculated using the following equation:

ITA° = [arctan(L* − 50)/b*)] × 180/π(1)

L* represents luminance, and its values range from black (0) to white (100). The values of b* range from yellow (−b*) to blue (+b*). 

The differences along the luminance axis and the yellow-blue axis determine the intensity of the skin pigmentation. In general, the higher the ITA°, the lighter the skin, thus the greater the lightening induced by the topical treatment.

**Figure 4:**
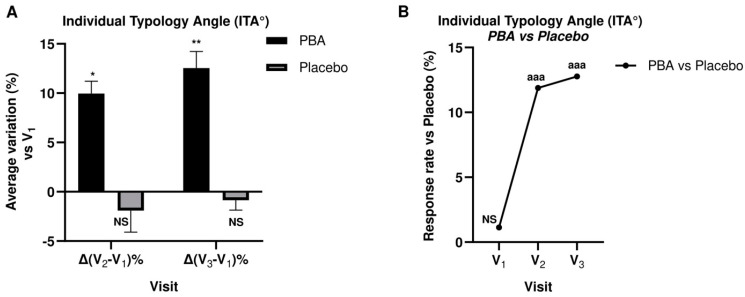
Individual Typology Angle (ITA°) during 30-day topical treatment with PBA or placebo. (**A**) ITA° average percentage variation vs. V_1_; (**B**) ITA° change vs. placebo before (V_1_), 15 days (V_2_) and 30 days (V_3_) after treatment with PBA. (NS: not significant; * *p* < 0.05, ** *p* < 0.01, Student *t*-test Vx vs. V_1_; ^aaa^ *p* < 0.001 ANOVA test PBA group vs. Placebo).

ITA° measurements were taken at the baseline t = 0 (V_1_), after 15 days (V_2_), and after 30 days (V_3_). The average ITA° values at V_1_, V_2_, and V_3_ measured on the PBA-treated areas were all compared with the corresponding points measured on the placebo-treated areas to yield the average differences Δ(V_2_ − V_1_) and Δ(V_3_ − V_2_) expressed as percentages. Figure 4 reports the results of the above measurements as histograms and scatter line.

ITA° values in the PBA group during the study were significantly increased (*p* < 0.05) after 15 and 30 days. Specifically, after 15 and 30 days of PBA topical application, the average percentage augmentation of ITA° increased by 10% and 13%, respectively. Conversely, placebo-treated subjects did not exhibit higher ITA° values, suggesting that the placebo treatment does not exert a lightening activity when applied topically on the skin. 

**Figure 5:**
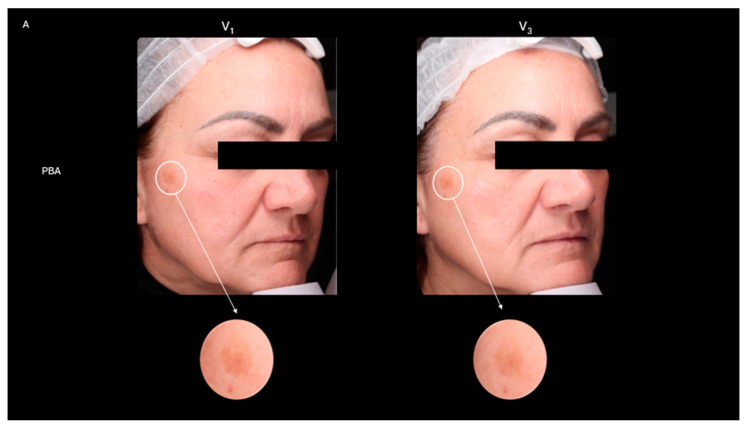

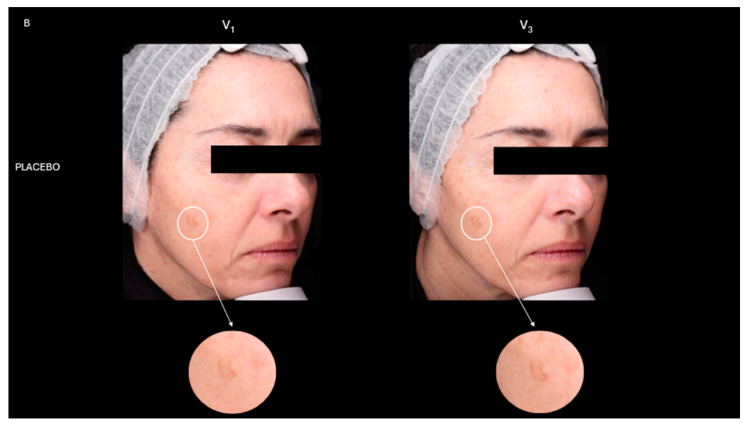
Skin brightening and whitening during 30-day topical treatment with (**A**) PBA and (**B**) Placebo before (V_1_) and 30 days (V_3_) after treatment.

Figure 5A,B shows pictures of panelists treated with PBA or placebo according to the assigned treatment group.

### 2.4. Skin Depigmentation Activity

We also analyzed the improvement of skin discoloration. Brown spots (Bs) were detected using RBX™ Technology of VISIA (Canfield Scientific Inc., Parsippany, NJ, USA), while UV spots were identified through the UV filter of the device. Bs represent lesions deeper within the skin, such as hyperpigmentation, freckles, lentigines, and melasma, while UV spots indicate superficial melanin accumulation after repeated sun exposure. 

After 15 days of PBA topical application (V_2_), the average decrease of Bs and UV spots was −9.2% (*p* < 0.05) and −7.3% (*p* < 0.01), respectively. Bs and UV spots further decreased at the end of the treatment (V_3_, Table 1). Placebo-treated subjects did not register lower UV spots or Bs values. The ANOVA test confirmed a statistical difference between the PBA and placebo topical treatments (*p* < 0.001). 

**Figure 6:**
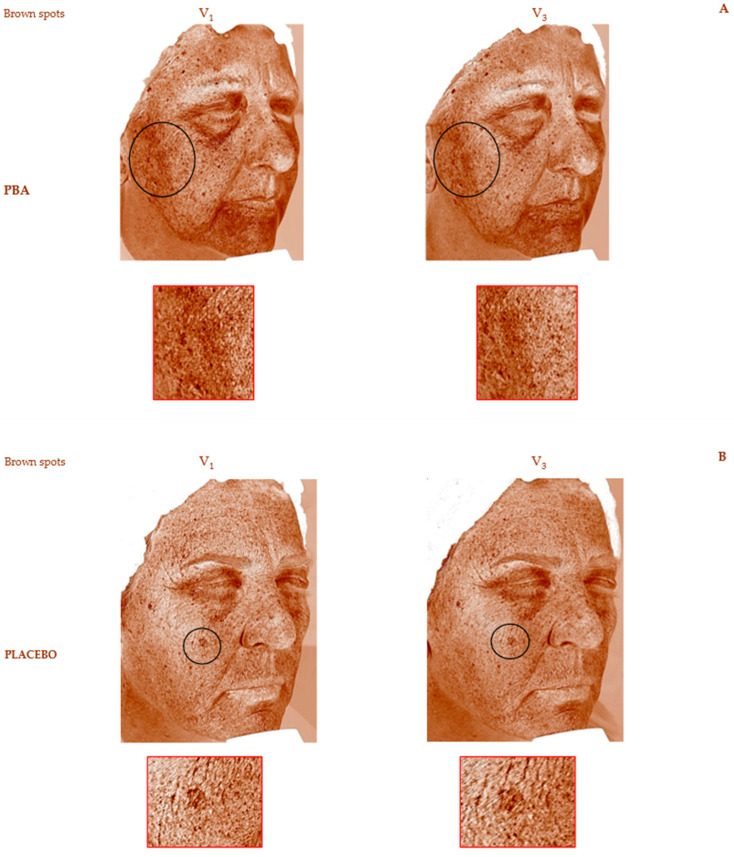
Skin depigmenting activity was assessed on brown spots during 30-day topical treatment with (**A**) PBA and (**B**) Placebo before (V_1_) and 30 days after treatment (V_3_).

**Figure 7:**
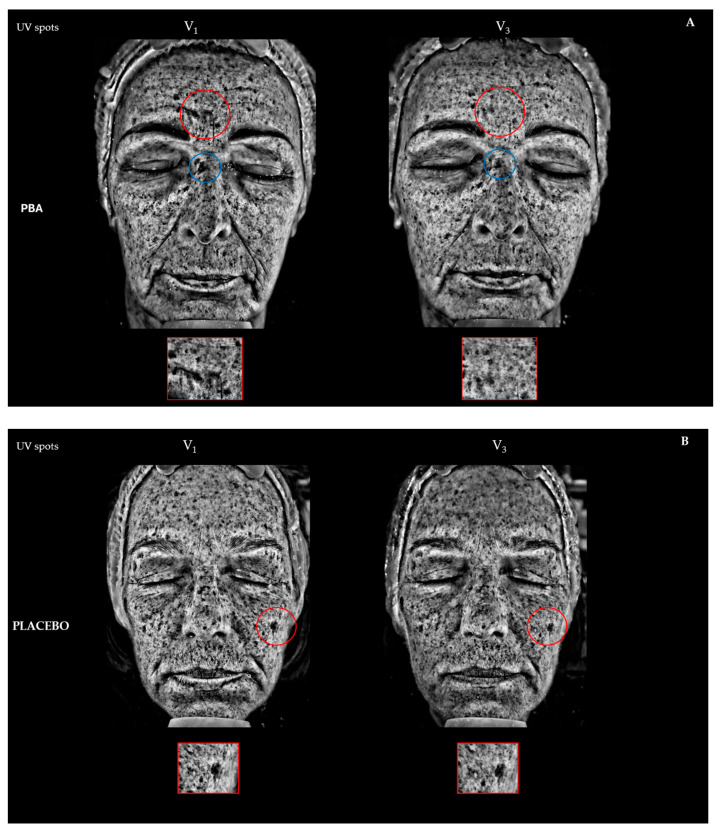
Skin photoaging reverse activity assessed on UV spots during 30-day topical treatment with (**A**) PBA and (**B**) Placebo before (V_1_) and 30 days after treatment (V_3_).

The VISIA^®^ Skin Analysis assesses the extent of aging and sun damage present in the deeper layers of the skin. We measured the presence of brown and UV spots. Brown spots, identified by RBX technology (Figure 6A,B), include skin lesions such as hyperpigmentation, freckles, lentigines, and melasma. These occur due to an excess of melanin, leading to an uneven skin tone. UV spots, on the other hand, form when melanin clumps beneath the skin’s surface due to sun damage. These spots are typically invisible under normal light but become visible through the selective absorption of UV light by epidermal melanin (Figure 7A,B). A higher number of Bs and UV spots and a corresponding higher score indicate more severe skin damage, underscoring the need for treatments or products to address sun damage and aging signs like uncontrolled melanogenesis. 

### 2.5. Skin Firmness and Elasticity (Suction Parameters Uf and Ua/Uf)

Skin Firmness and elasticity were measured at days 0 (V_1_), 15 (V_2_), and 30 (V_3_) for both treatment groups. The measured values and the changes from baseline (V_1_) are shown in Table 2. 

**Figure 8:**
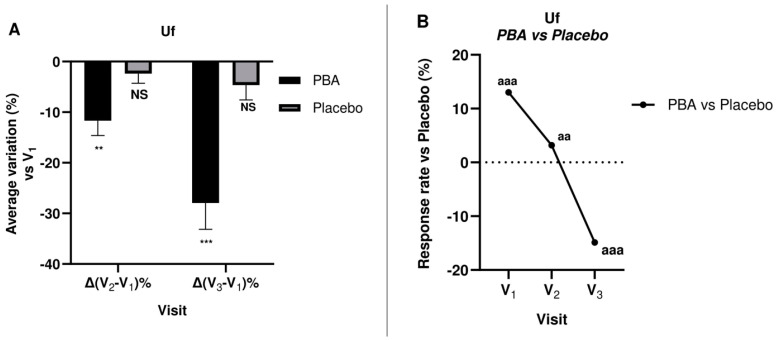
Skin firmness (Uf) during 30-day topical treatment with PBA or placebo. (**A**) Uf average percentage variation vs. V_1_; (**B**) Uf change vs. placebo before (V_1_), 15 days (V_2_), and 30 days (V_3_) after treatment with PBA. (NS: not significant; ** *p* < 0.01, *** *p* < 0.001 Student *t*-test Vx vs. V_1_; ^aa^ *p* < 0.01, ^aaa^ *p* < 0.001 ANOVA test PBA group vs. placebo).

**Figure 9:**
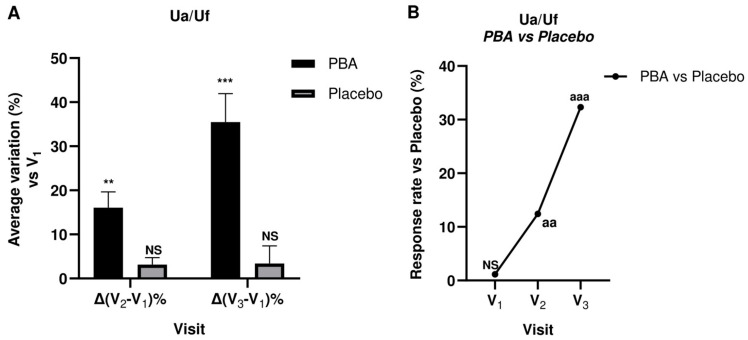
Skin elasticity (Ua/Uf) during 30-day topical treatment with PBA or placebo. (**A**) Ua/Uf average percentage variation vs. V_1_; (**B**) Ua/Uf change vs. placebo before (V_1_), 15 days (V_2_), and 30 days (V_3_) after treatment with PBA. (NS: not significant; ** *p* < 0.01, *** *p* < 0.001 Student *t*-test Vx vs. V_1_, ^aa^ *p* < 0.01, ^aaa^ *p* < 0.001 ANOVA test PBA group vs. placebo).

The measurements were carried out using a 2-mm probe, and a significant difference (*p* < 0.05) was found in PBA-treated subjects at all visits, whereas the placebo did not show the same efficacy. Furthermore, the ANOVA test indicated statistical differences between groups (Figure 8 and Figure 9).

To continue, an emulsion with 1.5% PBA significantly decreased Uf (R_0_—skin firmness) by 11.7% (V_2_) and 27.9% at V_3_. Furthermore, PBA significantly improved skin elasticity (Ua/Uf) by 16.1% at V_2_ and 35.5% after 30 days of treatment (V_3_). Therefore, we deduced that repeated topical applications of PBA ameliorated the panelists’ skin visco-elastic properties.

## 3. Discussion

Since cutaneous pigmentary heterogeneity and melasma is a widespread blemish affecting a large slice of the world population [33], skin-lightening molecules have drawn wide interest worldwide for their relevant applications in both cosmetic and dermatological fields [34,35,36,37,38]. Their use is widely diffused in cosmetic practice to improve skin appearance, but also in medical therapy for the treatment of hyper-pigmentary disorders such as melasma, café-au-lait spots and solar lentigo [39], the most dreaded aging and photo-damage signs, often accompanied by elastosis [40,41,42]. 

The formation of skin spots is mainly associated with an over-activation of melanogenesis, making its regulation a major target of lightening agents. Particularly, melanogenesis is controlled by several molecular modulators such as microphthalmia-associated transcription factor (MITF), tyrosinase, and tyrosinase-related proteins (TRP1 and TRP2). Short-chain fatty acids (SCFAs) are agonists of peroxisome proliferator-activated receptors (PPARs), which regulate various skin functions, including keratinocyte proliferation, wound healing, and melanogenesis. PPAR-γ agonists have been shown to reduce melanogenesis, thus acting as depigmenting agents in cosmetics [27]. The binding to PPARγ results in reduced tyrosinase mRNA expression, which in turn results in less tyrosinase formation, thus leading to reduced melanogenesis [28]. For these reasons, PPAR agonists showed potential for treating hyperpigmentation conditions like melanoma and melasma. Between SCFA’s PPAR agonists, butyrate can be included. Butyrate is produced by the bacterial fermentation of dietary fibers in the gut. It plays a crucial role in maintaining gut health and exhibits various beneficial effects on the body, including anti-inflammatory properties and regulation of the immune system. While butyrate is produced in abundance by gut flora, its production by skin flora is minimal. Despite its potential benefits for skin health, including promoting wound healing and modulating inflammation, butyrate is rarely used in topical applications due to its unpleasant rancid smell. 

This study investigated, for the first time, the tyrosinase inhibitory activity of PBA, an odorless butyrate derivative. Tyrosinase is a copper-containing protein that catalyzes phenolic oxidation of L-tyrosine, playing a critical role in melanin production. We evaluated tyrosinase inhibition by PBA and butyrate through in vitro assays on mushroom tyrosinase, which is widely used to search for tyrosinase inhibitors. PBA and butyrate showed IC_50_ values of 34.7 mM and 120.3 mM, respectively. Docking calculations using a homology model of human tyrosinase suggested that PBA may be endowed with inhibitory activity against this isoform.

The anti-aging and anti-spot efficacy of topical PBA was evaluated in a randomized, double-blind, parallel-arm, placebo-controlled clinical trial involving 43 women affected by photo-damage. The results of this study showed that PBA significantly improved skin conditions compared to the placebo and was well tolerated. Specifically, PBA demonstrated strong skin depigmenting activity on both UV and brown spots (UV: −12.7% and −9.9%, Bs: −20.8% and −17.7% after 15 and 30 days, respectively, *p* < 0.001). Moreover, PBA brightened and lightened the skin (ITA°: +12% and 13% after 15 and 30 days, respectively, *p* < 0.001).

Panelists also showed a significant improvement of their skin visco-elastic properties, such as firmness and elasticity. As is known, butyrate promotes the synthesis of hyaluronic acid by fibroblasts, boost collagen synthesis, and reduces the activity of protein kinase MEK1/2 [19]. All these activities may explain the improvement of the skin visco-elastic parameters. Specifically, PBA statistically increased skin elasticity (Ua/Uf) by 12.4% and 90.2% vs. placebo (*p* < 0.001) and improved skin firmness (V_2_: −11.7%, *p* < 0.05; V_3_: −27.9%, *p* < 0.01 vs. baseline).

In conclusion, PBA represents a promising therapeutic solution for treating hyperpigmentation conditions and counteracting the signs of photo-induced aging, offering significant clinical benefits and good tolerability. These results open the way for further research and development of even more effective compounds based on the PBA structure.

## 4. Materials and Methods

### 4.1. Tyrosinase Inhibition Assay

The inhibitory activity of tyrosinase was assessed using a modified dopachrome method, employing L-tyrosine as the substrate, based on the spectrophotometric detection of the quinone oxidation product recorded at 492 nm [43]. An amount of 70 µL of different concentrations of the samples was added to a 96-well microplate. Subsequently, 100 µL of 1 mM L-tyrosine and 30 µL of (*Agaricus bisporus*) mushroom tyrosinase 500 U/mL were added, mixed well, and incubated at room temperature for 30 min. Finally, absorbance was measured at 492 nm using a microplate reader. The control of the assay was prepared in parallel by replacing the volume of the sample with the same volume of buffer. 1 mM L-tyrosine and the mushroom tyrosinase (500 U/mL) were prepared in a 50 mM sodium phosphate buffer at a pH of 6.5. The analyzed samples were dissolved in 100% *v*/*v* DMSO and diluted with 50 mM sodium phosphate buffer pH 6.5 to obtain a final 1% *v*/*v* DMSO concentration in the enzymatic mixture. The percentage of tyrosinase inhibition was calculated as follows:

$$\%\;of\;inhibition=\left(\frac{Acontrol-Asample}{Acontrol}\right)*100$$

The inhibitory effect was expressed as the concentration required to inhibit tyrosinase activity by 50% (IC_50_). 

### 4.2. Molecular Docking Simulation

Molecular docking studies were conducted using Maestro Schrödinger suite version 2023–4 (Schrödinger, LLC, New York, NY, USA) on the recent available Swiss-model Expasy (https://swissmodel.expasy.org/, accessed on 26 June 2024) software-based homology model of human tyrosinase [44] built on the crystallographic structure of TYRP1 (PDB: 9YE8) exhibiting a high degree of similarity to the model predicted by the AlphaFold approach (AF-P14679-F1 model_v2). The grid box was defined using 9EY8 ligand following the close disposition to the metal centers in the active site of tyrosinase.

### 4.3. Clinical Study 

We designed a randomized, double-blind, placebo-controlled cosmetic clinical trial involving 43 healthy women aged 40–60 years with skin photo-damage, such as the presence of visible wrinkles and diffused hyperpigmentation. They were randomly divided into two groups, and then fully informed about the details and objectives of the study. Participation was voluntary and written informed consent was obtained from the enrolled participants. 

**Figure 10:**
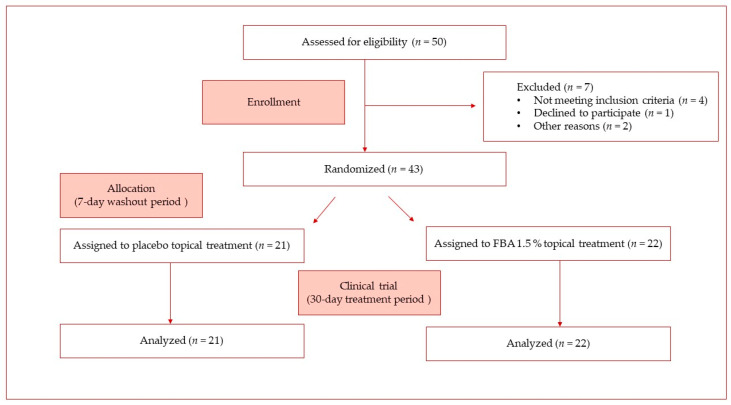
Clinical trial flowchart.

Participants also declared no allergies to commonly employed cosmetic ingredients. They were instructed to use approximately 2 mg per day of the assigned cosmetic *w*/*o* emulsions twice daily for 30 days, after a washout period of 7 days. Exclusion criteria were a history of dermatological disorders or pregnancy. Eligible subjects were randomly assigned either to the placebo-treated group (mean age 53.6 ± 3.1 years) or to the PBA-treated group (mean age 54.1 ± 2.4 years) and the study was conducted in double-blind. All skin parameters were detected on the right side of the face by the same technician, after a 30-min-conditioning procedure in a room with controlled humidity and temperature (50 ± 5% r.H. and 20 ± 2 °C). All of the measurements were performed in the morning, at the same time of the day, to maximally exclude the effect of the circadian rhythm. The measured skin parameters were as follows: skin elasticity and firmness, skin color and lightness, and skin spots. Measurements of the above skin parameters were performed every 2 weeks (V_2_, V_3_) after baseline records (V_1_). Figure 10 summarizes all the steps of the clinical study leading to the enrollment and allocation to the group treatment. As reported, no subjects dropped out of the study. Thus, 43 subjects were included in the final analysis. As it is preferable to evaluate changes in skin color and luminosity when there is low UV ray exposure, this test was conducted from November 2023 to January 2024.

Considering the cosmetic purpose of the present study and the targeting of healthy human subjects, a submission to the relevant ethics committee was not required. The clinical trial (EAE-23N02) was conducted at the University of Naples Federico II—RD Cosmetics laboratory, which fulfills a management system according to the UNI EN ISO 9001:2015 standards [45], certified by IMQ Spa (Milan, Italy). Nevertheless, the study strictly fulfilled the principles of the Helsinki Declaration and the COLIPA Guidelines for the evaluation of the efficiency of cosmetic products and the SCCS guidelines. Moreover, all the test subjects consented to the processing of their data and images taken during the trial.

All participants received product application training at the start of treatment and undertook the first application under supervision. At the initial visit (V_1_), the participants were examined independently by an instrumental analysis, and baseline photographs were taken. Participants were then reviewed on days 15 (visit 2, V_2_) and 30 (visit 3, V_3_), 3 h after they had washed their faces. Skin acceptability based on an occlusive patch test was assessed previously. 

### 4.4. PBA and Excipients

Phenylalanine butyramide (C13H18N2O2) (234.29 g/mol) was synthesized according to the patent US patent (US 2011 00983.19A1; 28 April 2011). All the ingredients employed for the topical formulation were cosmetic-grade and procured from ACEF Spa (Fiorenzuola D’arda, Piacenza, Italy).

### 4.5. PBA-Based Topical Preparation

Topical preparations *w*/*o* emulsions were prepared using a phase inversion technique [46]. Initially, oily constituents (Phase A), such as Polyglyceryl-4 Isostearate (4.00% *w*/*w*), Cera Alba (1.50% *w*/*w*), PEG-40 Hydrogenated Castor Oil (1.50% *w*/*w*), Ethylhexyl Palmitate (11.50% *w*/*w*), Caprylic/Capric Triglycerides (11.50% *w*/*w*), and the PBA (1.50% *w*/*w*) were mixed using a magnetic stirrer at 200 ± 25 rpm at 70 ± 5 °C. 

After the complete melting, the oil phase was mixed with the aqueous phase (Phase B), containing deionized water (60.90% *w*/*w*), Sodium Gluconate (0.20% *w*/*w*), Magnesium Sulfate heptahydrate (0.50% *w*/*w*) and Glycerin (3.00% *w*/*w*), previously heated to 70 ± 5 °C, using the mechanical stirrer of a Silverson L5T Laboratory Mixer (SBL, Shanghai, China), at 4500–5000 rpm until 50 ± 2 °C, and at 2500–3000 rpm until the mixture becomes smooth and homogenous. Once it reached 37 °C, the cream was mixed with the final phase (Phase C) containing deionized water (3.00% *w*/*w*) and phenoxyethanol dissolved in Ethylhexylglycerin (0.90% *w*/*w*), which constitutes the preservative system of the cosmetic formulation.

After 24 h, viscosity (25.532–26.718 MPa; spindle 64, 20 rpm, 22 °C) was measured with a Brookfield DV-E (Ametek Inc., Berwyn, PA, USA). 

Finally, the cosmetic emulsions were placed in anonymous 50 mL air-tight containers. The placebo emulsion was prepared analogously and contained all the listed ingredients (Table 3) except for the PBA.

### 4.6. Skin Parameters

The cutaneous parameters best suited to assess the product’s ability to give skin a younger and healthier appearance by acting on the most common signs of aging and satisfying the newest general demand for easily obtainable multi-functional active ingredients are reported as follows:

- Skin complexion color was measured as Individual Typology Angle (ITA°) values with the Skin-colorimeter CL 400 (Courage + Khazaka Electronic GmbH, Köln, Germany).
- Severity of skin pigmentation, sun damage, and premature aging were investigated through brown and UV-spot detection using VISIA 7th (Canfield Scientific Inc., Parsippany, NJ, USA) photographic analysis [47,48] via RBX technology (Red/Brown Subsurface Analysis) [49,50].
- Before and after photos were also collected to compare results.

In conclusion, because of sun damage, skin undergoes premature aging, with manifestations of elastosis and collagenosis [51,52,53,54,55] such as the degradation of the dermis collagen and elastin. Since, as anticipated, BA can promote the synthesis of hyaluronic acid in fibroblasts, boost collagen biosynthesis, and reduce the activity of the MEK 1/2 and MAPK, it could also be able to induce changes in the visco-elastic properties of the skin. For this reason, as a secondary endpoint, the following skin parameters were observed throughout the study period:

- Skin firmness and elasticity were evaluated by the Cutometer^®^ DUAL MPA 580 (Courage + Khazaka electronic GmbH, Köln, Germany) with a standard probe (Ø mm^2^). The surface of the skin is sucked into the probe opening and a constant 450 mbar vacuum is applied for a preset time of 5 seconds, after which the skin is released following 5 seconds of air depression break (relaxation time). Thus, the skin can return to its original position. Five suction cycles (1 cycle: suction/release) are performed on the same point. The optical detection system inside the probe measured several skin features, such as the penetration depth, the resistance to the negative pressure, which is related to the skin’s firmness, and the ability of the skin to return to its original state (skin elasticity). The optical detection system includes a light source and a light receptor, along with two prisms facing each other, which project the light from transmitter to receptor. During real-time skin measurements, the light intensity changes depending on the skin penetration depth and relaxation time. These data are displayed as curves, which report the height (in mm) reached by the skin during the suction phase and the different levels of “skin return” during the relaxation time in real-time measurement procedures, allowing the calculation of the skin’s visco-elastic properties and skin aging. As described by Dobrev et al. [54], the following regions can be seen in a typical skin deformation curve: Ue, immediate distension; Uv, delayed distension; Uf, final distension; Ur, immediate retraction; and Ua, final retraction. The skin mechanical parameters considered in this study were skin firmness (Uf) and skin elasticity (Ua/Uf).

### 4.7. Data Analysis and Statistics

All results are presented as the change from baseline (i.e., before cosmetic formulation application, V_1_ values), and the placebo group was used as the control. All data were subjected to multigroup comparison tests. When *p* < 0.05, a Student *t*-test confirmed the average value differences from baseline (V_1_), whereas the ANOVA test was employed to investigate intergroup differences (vs. placebo). All data were analyzed using SPSS V.28 software, setting: V_1_ = average baseline value; V_2_ = average recorded value after 15-day treatment; and V_3_ = average recorded value after 30 days of treatment (Tables S1–S15).

## Figures and Tables

**Table 1:** Effect of 1.5% PBA topical application on skin hyperpigmentation severity (Brown and UV Spots). (* *p* < 0.05, ** *p* < 0.01 Student *t*-test Vx vs. V_1_; ^aaa^ *p* < 0.001 ANOVA test PBA group vs. Placebo).

Placebo		PBA		
Brown spots (Mean ± SD)		Change vs. V_1_ ± SEM		Change vs. V_1_ ± SEM
V_1_	21.5 ± 3.6	/	21.5 ± 3.1	/
V_2_	23.5 ± 4.0	10.4 ± 0.7	19.4 ± 2.5	−9.2 ± 0.4 * ^aaa^
V_3_	22.7 ± 3.6	6.4 ± 0.6	19.3 ± 3.6	−10.9 ± 0.4 * ^aaa^
UV spots (Mean ± SD)		Change vs. V_1_ ± SEM		Change vs. V_1_ ± SEM
V_1_	24.6 ± 2.9	/	24.4 ± 2.1	/
V_2_	25.4 ± 2.5	3.6 ± 0.4	22.5 ± 1.5	−7.3 ± 0.3 ** ^aaa^
V_3_	22.8 ± 1.6	1.7 ± 0.2	22.8 ± 1.6	−6.7 ± 0.2 ** ^aaa^

At visits 1, 2, and 3 of the PBA topical application period, skin hyperpigmentation severity was measured using high-resolution photos of the panelists’ faces. The changes in values for the skin parameters relative to the baseline condition were determined using VISIA 7th (Canfield Scientific Inc., Parsippany, NJ, USA). Mean value ± SD at visits 1, 2 and 3 and the changes ± SEM from day 0 (V_1_) to day 30 (V_3_) are presented. Comparisons to the placebo group were carried out using an ANOVA test.

**Table 2:** Effect of 1.5% PBA topical application on skin firmness and elasticity (2-mm probe). (* *p* < 0.05, ** *p* < 0.01, *** *p* < 0.001 Student-*t* test Vx vs. V_1_; ^aa^ *p* < 0.01, ^aaa^ *p* < 0.001 ANOVA test PBA group vs. Placebo).

Placebo		PBA		
Uf (Mean ± SD)		Change vs. V_1_ ± SEM		Change vs. V_1_ ± SEM
V_1_	0.270 ± 0.034	/	0.310 ± 0.042	/
V_2_	0.264 ± 0.049	−2.4 ± 0.2	0.273 ± 0.052	−11.7 ± 0.3 * ^aa^
V_3_	0.259 ± 0.061	−4.7 ± 0.4	0.226 ± 0.084	−27.9 ± 0.6 *** ^aaa^
Ua/Uf (Mean ± SD)		Change vs. V_1_ ± SEM		Change vs. V_1_ ± SEM
V_1_	0.510 ± 0.055	/	0.506 ± 0.056	/
V_2_	0.592 ± 0.104	0.012 ± 0.037	0.585 ± 0.106	16.1 ± 0.4 ** ^aa^
V_3_	0.687 ± 0.148	0.018 ± 0.094	0.678 ± 0.152	35.5 ± 0.8 *** ^aaa^

At visit 1, 2, and 3 of the PBA topical application period, skin elasticity and firmness at the cheeks were measured. The changes in values for the skin parameters relative to the baseline condition (V_1_), as determined using the 2-mm probe. Mean value ± SD at visits 1, 2, and 3 and the changes from day 0 to day 30 are presented. Comparisons to the placebo group were carried out using an ANOVA test.

**Table 3:** Composition of PBA-based *w*/*o* emulsion.

Phase	Ingredients RB ^1^ = 5.00	Function	% *w*/*w*
A	Polyglyceryl-4 Isostearate	Emulsifier	4.00
A	Cera Alba	Consistency factor	1.50
A	PEG-40 Hydrogenated Castor Oil	Consistency factor	1.50
A	Ethylhexyl Palmitate	Emollient	11.50
A	Caprylic/Capric Triglycerides	Emollient	11.50
A	PBA	Active ingredient	1.50
B	Aqua	Solvent	Qs ^2^ to 100
B	Sodium Gluconate	Chelating agent	0.20
B	Magnesium Sulfate heptahydrate	Viscosizing agent	0.50
B	Glycerin	Humectant	3.00
C	Aqua	Solvent	3.00
C	Phenoxyethanol (and) Ethylhexylglycerin	Preservative	0.90

^1^ Required HLB value; ^2^ quantity sufficient.

## Data Availability

Data is contained within the article and Supplementary Materials.

## Supplementary Materials

The following supporting information can be downloaded at: https://www.mdpi.com/article/10.3390/ijms25137310/s1.
