# Supplementary material for: Phenylalanine Butyramide: A Butyrate Derivative as a Novel Inhibitor of Tyrosinase
Source: Int J Mol Sci. 2024 Jul 3;25(13):7310. doi: 10.3390/ijms25137310 (PMC11242249; doi:10.3390/ijms25137310)
Supplement: Supplementary file 1 [file ijms-25-07310-s001.zip › ijms-3062353-supplementary.pdf]

International Journal of Molecular Sciences

MDPI

Phenylalanine Butyramide: a Butyrate Derivative as a Novel Inhibitor of Tyrosinase

Ritamaria Di Lorenzo [1], Vincenzo Di Lorenzo [2], Teresa Di Serio [1], Adua Marzocchi [1], Lucia Ricci [1], Eleonora Vardaro [1], Giovanni Greco [1], Maria Maisto [1], Lucia Grumetto [1], Vincenzo Piccolo [1], Elena Morelli [1] and Sonia Laneri [1,*]

Table S1. Effect of 1.5% w/w PBA on brown spots of treated volunteers at V1, V2 and V3. Brown Spots value for each volunteer and their variation are reported, along with average percentage variation value ± SD.

Brown Spots V1 – V2 1.5% w/w PBA				
# Study subject	V1	V2	V2-V1	Δ %
1	23,5	17,9	-5,6	-23,7
2	22,8	22,6	-0,2	-0,9
3	22,0	21,7	-0,3	-1,4
4	15,9	14,5	-1,4	-8,8
5	16,1	15,1	-1,0	-6,2
6	23,9	18,8	-5,1	-21,3
7	18,0	17,3	-0,7	-3,9
8	18,0	17,7	-0,3	-1,7
9	26,8	20,3	-6,5	-24,3
10	21,3	21,1	-0,2	-0,9
11	22,6	22,2	-0,4	-1,8
12	24,1	20,8	-3,3	-13,7
13	25,4	23,3	-2,1	-8,3
14	26,7	23,9	-2,8	-10,5
15	20,3	19,7	-0,6	-3,0
16	20,6	19,2	-1,4	-6,8
17	18,5	18,5	0,0	0,0
18	19,5	18,0	-1,5	-7,7
19	21,0	19,2	-1,8	-8,6
20	20,0	17,0	-3,0	-15,0
21	23,0	20,1	-2,9	-12,6
22	24,1	18,8	-5,3	-22,0
**Average**	21,5	19,4	-2,1	**-9,2**
**Std. Dev**	3,1	2,5	2,0	7,9
N. study subjects	22	22	22	
t-test	2,50			
Degree of freedom	42			
p	0,016393			

Brown Spots V1 – V3 1.5% w/w PBA				
# Study subject	V1	V3	V3-V1	Δ %
1	23,5	20,0	-3,5	-14,7
2	22,8	22,8	0,0	0,0
3	22,0	21,1	-0,9	-4,1
4	15,9	14,3	-1,6	-10,1
5	16,1	12,1	-4,0	-24,8
6	23,9	21,1	-2,8	-11,7
7	18,0	14,2	-3,8	-21,1
8	18,0	16,1	-1,9	-10,6
9	26,8	18,2	-8,6	-32,1
10	21,3	20,1	-1,2	-5,6
11	22,6	21,3	-1,3	-5,8
12	24,1	22,7	-1,4	-5,8
13	25,4	23,1	-2,3	-9,1
14	26,7	25,5	-1,2	-4,5
15	20,3	20,0	-0,3	-1,5
16	20,6	17,3	-3,3	-16,0
17	18,5	14,2	-4,3	-23,2
18	19,5	14,4	-5,1	-26,2
19	21,0	20,4	-0,6	-2,9
20	20,0	20,3	0,3	1,5
21	23,0	23,5	0,5	2,2
22	24,1	20,8	-3,3	-13,7
**Average**	21,5	19,3	-2,3	**-10,9**
**Std. Dev**	3,1	3,6	2,1	9,6
N. study subjects	22	22	22	
t-test	2,25			
Degree of freedom	42			
p	0,02957530			

International Journal of Molecular Sciences

MDPI

Table S2. Effect of placebo on brown spots of treated volunteers at V1, V2 and V3. Brown Spots value for each volunteer and their variation are reported, along with average percentage variation value ± SD.

Brown Spots V1 – V3 PLACEBO				
**# Study subject**	**V1**	**V2**	**V2-V1**	**Δ %**
1	21,0	21,7	0,7	3,3
2	21,7	28,6	6,9	31,8
3	26,8	29,1	2,3	8,6
4	17,8	23,2	5,4	30,3
5	20,8	22,5	1,7	8,2
6	21,7	22,6	0,9	4,1
7	15,9	22,5	6,6	41,5
8	11,8	14,3	2,5	21,2
9	19,6	22,5	2,9	14,8
10	21,3	25,7	4,4	20,7
11	24,3	28,3	4,0	16,5
12	24,4	22,7	-1,7	-7,0
13	26,8	29,0	2,2	8,2
14	23,4	20,1	-3,3	-14,1
15	21,4	21,0	-0,4	-1,9
16	19,0	18,1	-0,9	-4,7
17	24,8	23,9	-0,9	-3,6
18	19,1	19,8	0,7	3,7
19	25,2	29,8	4,6	18,3
20	21,0	25,7	4,7	22,2
21	23,0	22,0	-1,0	-4,3
**Average**	21,5	23,5	2,0	**10,4**
**Std. Dev**	3,6	4,0	2,8	14,4
N. study subjects	21	21	21	
t-test	1,721478			
Degree of freedom	40			
p	0,092891			

Brown Spots V1 – V3 PLACEBO				
**# Study subject**	**V1**	**V3**	**V3-V1**	**Δ %**
1	21,0	20,5	-0,5	-2,4
2	21,7	24,1	2,4	11,1
3	26,8	22,4	-4,4	-16,4
4	17,8	19,4	1,6	9,0
5	20,8	21,3	0,5	2,4
6	21,7	23,6	1,9	8,8
7	15,9	16,4	0,5	3,1
8	11,8	15,2	3,4	28,8
9	19,6	17,1	-2,5	-12,8
10	21,3	26,0	4,7	22,1
11	24,3	27,2	2,9	11,9
12	24,4	24,8	0,4	1,6
13	26,8	26,6	-0,2	-0,7
14	23,4	25,9	2,5	10,7
15	21,4	22,0	0,6	2,8
16	19,0	24,5	5,5	28,9
17	24,8	24,5	-0,3	-1,2
18	19,1	18,6	-0,5	-2,6
19	25,2	27,9	2,7	10,7
20	21,0	24,9	3,9	18,4
21	23,0	23,0	0,0	0,0
**Average**	21,5	22,7	1,2	**6,4**
**Std. Dev**	3,6	3,6	2,3	11,7
N. study subjects	21	21	21	
t-test	1,06954032			
Degree of freedom	40			
p	0,29123813			

Table S3. Effect of 1.5% w/w PBA on individual typology angle of treated volunteers at V1, V2 and V3. Individual Typology Angle value for each volunteer and their variation are reported, along with average percentage variation value ± SD.

Individual Typology Angle V1 – V2 1.5% w/w PBA				
**# Study subject**	**V1**	**V2**	**V2-V1**	**Δ %**
1	38	42	4	11
2	49	58	9	18
3	32	39	7	22
4	41	42	1	2
5	37	40	3	7
6	44	51	7	15
7	39	42	3	8
8	39	40	1	3
9	46	50	4	10
10	51	54	3	6
11	38	43	5	13
12	43	47	4	10
13	43	50	7	16
14	48	50	2	4
15	30	33	3	10
16	31	34	3	10
17	35	39	4	11
18	41	49	8	20
19	36	41	5	14
20	41	42	1	2
21	43	44	1	2
22	44	46	2	5
**Average**	40	44	4	**10**
**Std. Dev**	6	6	2	6
N. study subjects	22	22	22	
t-test	2,21			
Degree of freedom	42			
p	0,032427			

Individual Typology Angle V1 – V3 1.5% w/w PBA				
**# Study subject**	**V1**	**V3**	**V3-V1**	**Δ %**
1	38	40	2	5
2	49	60	11	22
3	32	41	9	28
4	41	52	11	27
5	37	44	7	18
6	44	48	4	9
7	39	40	1	3
8	39	46	7	18
9	46	48	2	5
10	51	55	4	8
11	38	43	5	13
12	43	46	3	7
13	43	45	2	5
14	48	50	2	4
15	30	33	3	10
16	31	37	6	19
17	35	42	7	20
18	41	44	3	7
19	36	40	4	11
20	41	43	2	5
21	43	53	10	23
22	44	48	4	9
**Average**	40	45	5	**13**
**Std. Dev**	6	6	3	8
N. study subjects	22	22	22	
t-test	2,78			
Degree of freedom	42			
p	0,00807118			

International Journal of Molecular Sciences

MDPI

Table S4. Effect of placebo on individual typology angle of treated volunteers at V1, V2 and V3. Individual Typology Angle value for each volunteer and their variation are reported, along with average percentage variation value ± SD.

Individual Typology Angle V1 – V3 PLACEBO				
**# Study subject**	**V1**	**V2**	**V2-V1**	**Δ %**
1	46	44	-2	-4
2	39	33	-6	-15
3	36	32	-4	-11
4	32	42	10	31
5	40	41	1	3
6	32	29	-3	-9
7	37	40	3	8
8	35	31	-4	-11
9	43	40	-3	-7
10	50	53	3	6
11	48	43	-5	-10
12	49	49	0	0
13	34	30	-4	-12
14	39	38	-1	-3
15	37	39	2	5
16	44	41	-3	-7
17	46	46	0	0
18	42	40	-2	-5
19	33	35	2	6
20	34	33	-1	-3
21	43	42	-1	-2
**Average**	40	39	-1	**-2**
**Std. Dev**	6	6	4	10
N. study subjects	21	21	21	
t-test	0,459294			
Degree of freedom	40			
p	0,648509			

Individual Typology Angle V1 – V3 PLACEBO				
**# Study subject**	**V1**	**V3**	**V3-V1**	**Δ %**
1	46	47	1	2
2	39	35	-4	-10
3	36	37	1	3
4	32	33	1	3
5	40	42	2	5
6	32	30	-2	-6
7	37	37	0	0
8	35	35	0	0
9	43	42	-1	-2
10	50	50	0	0
11	48	45	-3	-6
12	49	48	-1	-2
13	34	31	-3	-9
14	39	39	0	0
15	37	38	1	3
16	44	44	0	0
17	46	45	-1	-2
18	42	42	0	0
19	33	36	3	9
20	34	32	-2	-6
21	43	43	0	0
**Average**	40	40	0	**-1**
**Std. Dev**	6	6	2	5
N. study subjects	21	21	21	
t-test	0,21295131			
Degree of freedom	40			
p	0,83244655			

Table S5. Effect of 1.5% w/w PBA on UV spots of treated volunteers at V1, V2 and V3. UV spots value for each volunteer and their variation are reported, along with average percentage variation value ± SD.

UV Spots V1 – V2 1.5% w/w PBA				
**# Study subject**	**V1**	**V2**	**V2-V1**	**Δ %**
1	25,4	22,0	-3,4	-13,4
2	22,8	20,4	-2,4	-10,5
3	28,9	23,6	-5,3	-18,3
4	27,3	22,5	-4,8	-17,6
5	22,5	20,9	-1,6	-7,2
6	20,7	19,9	-0,8	-3,9
7	22,8	23,0	0,2	0,8
8	27,0	23,6	-3,4	-12,6
9	25,6	21,6	-4,0	-15,6
10	24,4	21,5	-2,9	-11,9
11	23,9	23,3	-0,6	-2,3
12	24,2	24,8	0,6	2,4
13	22,5	23,5	1,0	4,4
14	25,0	22,0	-3,0	-12,0
15	27,6	23,6	-4,0	-14,5
16	26,2	25,1	-1,1	-4,2
17	22,2	22,6	0,4	1,8
18	23,8	24,9	1,1	4,6
19	23,5	20,4	-3,1	-13,2
20	22,1	20,5	-1,6	-7,2
21	22,8	22,0	-0,8	-3,5
22	25,0	23,3	-1,7	-6,8
**Average**	24,4	22,5	-1,9	**-7,3**
**Std. Dev**	2,1	1,5	1,9	7,2
N. study subjects	22	22	22	
t-test	3,39			
Degree of freedom	42			
p	0,001528			

UV Spots V1 – V3 1.5% w/w PBA				
**# Study subject**	**V1**	**V3**	**V3-V1**	**Δ %**
1	25,4	23,1	-2,3	-9,1
2	22,8	22,8	0,0	0,0
3	28,9	23,6	-5,3	-18,3
4	27,3	23,3	-4,0	-14,7
5	22,5	20,6	-1,9	-8,6
6	20,7	19,5	-1,2	-5,7
7	22,8	23,0	0,2	0,8
8	27,0	25,8	-1,2	-4,6
9	25,6	24,8	-0,8	-3,1
10	24,4	22,3	-2,1	-8,4
11	23,9	21,8	-2,1	-8,9
12	24,2	22,8	-1,4	-5,6
13	22,5	21,0	-1,5	-6,6
14	25,0	23,2	-1,8	-7,3
15	27,6	25,7	-1,9	-6,9
16	26,2	23,1	-3,1	-11,9
17	22,2	23,9	1,7	7,6
18	23,8	21,9	-1,9	-8,0
19	23,5	20,8	-2,7	-11,5
20	22,1	20,3	-1,8	-8,1
21	22,8	21,5	-1,3	-5,7
22	25,0	24,1	-0,9	-3,6
**Average**	24,4	22,7	-1,7	**-6,7**
**Std. Dev**	2,1	1,7	1,4	5,4
N. study subjects	22	22	22	
t-test	2,98			
Degree of freedom	42			
p	0,00478720			

International Journal of Molecular Sciences

Table S6. Effect of placebo on UV spots of treated volunteers at V1, V2 and V3. UV Spots value for each volunteer and their variation are reported, along with average percentage variation value ± SD.

UV Spots V1 – V3 PLACEBO				
# Study subject	V1	V2	V2-V1	Δ %
1	21,3	23,1	1,8	8,5
2	25,7	26,0	0,3	1,2
3	26,1	25,2	-0,9	-3,4
4	21,1	23,2	2,1	10,0
5	27,2	27,5	0,3	1,1
6	28,2	26,3	-1,9	-6,7
7	20,7	24,6	3,9	18,8
8	26,5	25,2	-1,3	-4,9
9	23,3	27,1	3,8	16,3
10	29,3	26,9	-2,4	-8,2
11	22,4	22,7	0,3	1,3
12	22,1	23,4	1,3	5,9
13	23,5	23,3	-0,2	-0,9
14	26,7	29,5	2,8	10,5
15	22,6	22,0	-0,6	-2,7
16	26,2	25,4	-0,8	-3,1
17	28,3	28,3	0,0	0,0
18	25,3	28,6	3,3	13,0
19	21,2	24,7	3,5	16,5
20	28,2	29,4	1,2	4,3
21	20,5	20,3	-0,2	-1,0
**Average**	24,6	25,4	0,8	**3,6**
**Std. Dev**	2,9	2,5	1,9	8,0
N. study subjects	21	21	21	
t-test	0,926993			
Degree of freedom	40			
p	0,35949			

UV Spots V1 – V3 PLACEBO				
# Study subject	V1	V3	V3-V1	Δ %
1	21,3	22,1	0,8	3,8
2	25,7	25,4	-0,3	-1,2
3	26,1	26,8	0,7	2,7
4	21,1	21,9	0,8	3,8
5	27,2	28,3	1,1	4,0
6	28,2	26,2	-2,0	-7,1
7	20,7	23,5	2,8	13,5
8	26,5	25,5	-1,0	-3,8
9	23,3	21,8	-1,5	-6,4
10	29,3	29,5	0,2	0,7
11	22,4	22,7	0,3	1,3
12	22,1	23,2	1,1	5,0
13	23,5	24,2	0,7	3,0
14	26,7	26,0	-0,7	-2,6
15	22,6	23,2	0,6	2,7
16	26,2	27,3	1,1	4,2
17	28,3	27,7	-0,6	-2,1
18	25,3	26,3	1,0	4,0
19	21,2	21,6	0,4	1,9
20	28,2	28,1	-0,1	-0,4
21	20,5	22,2	1,7	8,3
**Average**	24,6	24,9	0,3	**1,7**
**Std. Dev**	2,9	2,5	1,1	4,7
N. study subjects	21	21	21	
t-test	0,40616413			
Degree of freedom	40			
p	0,68678682			

Table S7. Effect of 1.5% w/w PBA on Ua/Uf 2-mm probe of treated volunteers at V1, V2 and V3. Ua/Uf 2-mm probe value for each volunteer and their variation are reported, along with average percentage variation value ± SD.

Ua/Uf 2-mm probe V2 vs. V1 PBA group				
# Subject	V1	V2	Δ(V2 - V1)	Δ(V2 -V1)%
1	0,556	0,597	0,041	7,4
2	0,421	0,474	0,053	12,6
3	0,473	0,456	-0,017	-3,6
4	0,597	0,887	0,290	48,6
5	0,554	0,544	-0,010	-1,8
6	0,550	0,553	0,003	0,5
7	0,500	0,541	0,041	8,2
8	0,538	0,566	0,028	5,2
9	0,448	0,755	0,307	68,5
10	0,505	0,632	0,127	25,1
11	0,402	0,455	0,053	13,2
12	0,496	0,521	0,025	5,0
13	0,561	0,721	0,160	28,5
14	0,523	0,588	0,065	12,4
15	0,544	0,575	0,031	5,7
16	0,552	0,668	0,116	21,0
17	0,436	0,512	0,076	17,4
18	0,566	0,577	0,011	1,9
19	0,459	0,539	0,080	17,4
20	0,448	0,529	0,081	18,1
21	0,521	0,660	0,139	26,7
22	0,578	0,669	0,091	15,7
**Average**	0,510	0,592	0,081	**16,1**
**Std. Dev**	0,055	0,104	0,085	16,7
Nr of Subjects	22	22	22	
t-test	3,2502294			
degree of freedom	42			
p	0,0022744			

Ua/Uf 2-mm probe V3 vs. V1 PBA group				
# Subject	V1	V3	Δ(V3 - V1)	Δ(V3 -V1)%
1	0,556	0,447	-0,11	-19,60
2	0,421	0,604	0,18	43,47
3	0,473	0,412	-0,06	-12,90
4	0,597	0,695	0,10	16,42
5	0,554	0,810	0,26	46,21
6	0,550	0,643	0,09	16,91
7	0,500	0,631	0,13	26,20
8	0,538	0,571	0,03	6,13
9	0,448	0,440	-0,01	-1,79
10	0,505	0,509	0,00	0,79
11	0,402	0,712	0,31	77,11
12	0,496	0,686	0,19	38,31
13	0,561	0,786	0,23	40,11
14	0,523	0,889	0,37	69,98
15	0,544	0,665	0,12	22,24
16	0,552	0,887	0,34	60,69
17	0,436	0,702	0,27	61,01
18	0,566	0,691	0,13	22,08
19	0,459	0,722	0,26	57,30
20	0,448	0,856	0,41	91,07
21	0,521	0,883	0,36	69,48
22	0,578	0,864	0,29	49,48
**Average**	0,510	0,687	0,176	**35,5**
**Std. Dev**	0,055	0,148	0,146	30,3
Nr of Subjects	22	22	22	
t-test	5,2396136			
degree of freedom	42			
p	4,874E-06			

International Journal of Molecular Sciences

MDPI

Table S8. Effect of placebo on Ua/Uf 2-mm probe of treated volunteers at V1, V2 and V3. Ua/Uf 2-mm probe value for each volunteer and their variation are reported, along with average percentage variation value ± SD.

Ua/Uf 2-mm probe V2 vs. V1 Placebo group				
# Subject	V1	V2	Δ(V2 - V1)	Δ(V2 -V1)%
1	0,561	0,570	0,009	1,6
2	0,438	0,432	-0,006	-1,4
3	0,535	0,564	0,029	5,4
4	0,587	0,542	-0,045	-7,7
5	0,404	0,436	0,032	7,9
6	0,541	0,544	0,003	0,6
7	0,578	0,584	0,006	1,0
8	0,491	0,541	0,050	10,2
9	0,531	0,566	0,035	6,6
10	0,448	0,457	0,009	2,0
11	0,551	0,625	0,074	13,4
12	0,585	0,521	-0,064	-10,9
13	0,574	0,545	-0,029	-5,1
14	0,489	0,535	0,046	9,4
15	0,425	0,453	0,028	6,6
16	0,566	0,528	-0,038	-6,7
17	0,455	0,436	-0,019	-4,2
18	0,411	0,469	0,058	14,1
19	0,475	0,526	0,051	10,7
20	0,513	0,568	0,055	10,7
21	0,435	0,444	0,009	2,1
**Average**	0,504	0,518	0,014	**3,2**
**Std. Dev**	0,062	0,057	0,037	7,2
Nr of Subjects	21	21	21	
t-test	0,7585139			
degree of freedom	40			
p	0,4525898			

Ua/Uf 2-mm probe V3 vs. V1 Placebo group				
# Subject	V1	V3	Δ(V3 - V1)	Δ(V3 -V1)%
1	0,561	0,527	-0,034	-6,1
2	0,438	0,562	0,124	28,3
3	0,535	0,674	0,139	26,0
4	0,587	0,528	-0,059	-10,1
5	0,404	0,436	0,032	7,9
6	0,541	0,454	-0,087	-16,1
7	0,578	0,596	0,018	3,1
8	0,491	0,592	0,101	20,6
9	0,531	0,536	0,005	0,9
10	0,448	0,482	0,034	7,6
11	0,551	0,661	0,110	20,0
12	0,585	0,455	-0,130	-22,2
13	0,574	0,563	-0,011	-1,9
14	0,489	0,456	-0,033	-6,7
15	0,425	0,289	-0,136	-32,0
16	0,566	0,779	0,213	37,6
17	0,455	0,569	0,114	25,1
18	0,411	0,401	-0,010	-2,4
19	0,475	0,517	0,042	8,8
20	0,513	0,523	0,010	1,9
21	0,435	0,354	-0,081	-18,6
**Average**	0,504	0,522	0,017	**3,4**
**Std. Dev**	0,062	0,111	0,092	18,2
Nr of Subjects	21	21	21	
t-test	0,6215403			
degree of freedom	40			
p	0,5377706			

Table S9. Effect of 1.5% w/w PBA on Uf 2-mm probe of treated volunteers at V1, V2 and V3. Uf 2-mm probe value for each volunteer and their variation are reported, along with average percentage variation value ± SD.

Uf 2-mm probe V2 vs. V1 (baseline) PBA group				
# Subject	V1	V2	Δ(V2 - V1)	Δ(V2 -V1)%
1	0,330	0,280	-0,050	-15,2
2	0,220	0,252	0,032	14,5
3	0,375	0,368	-0,007	-1,9
4	0,246	0,158	-0,088	-35,8
5	0,390	0,285	-0,105	-26,9
6	0,330	0,308	-0,022	-6,7
7	0,275	0,276	0,001	0,4
8	0,305	0,286	-0,019	-6,2
9	0,329	0,378	0,049	14,9
10	0,306	0,181	-0,125	-40,8
11	0,289	0,254	-0,035	-12,1
12	0,326	0,285	-0,041	-12,6
13	0,266	0,239	-0,027	-10,2
14	0,278	0,254	-0,024	-8,6
15	0,336	0,269	-0,067	-19,9
16	0,277	0,251	-0,026	-9,4
17	0,289	0,264	-0,025	-8,7
18	0,299	0,250	-0,049	-16,4
19	0,323	0,218	-0,105	-32,5
20	0,356	0,321	-0,035	-9,8
21	0,308	0,299	-0,009	-2,9
22	0,369	0,332	-0,037	-10,0
**Average**	0,310	0,273	-0,037	**-11,7**
**Std. Dev**	0,042	0,052	0,042	13,801
Nr of Subjects	22	22	22	
t-test	2,6023123			
degree of freedom	40			
p	0,0129235			

Uf 2-mm probe V3 vs. V1 (baseline) PBA group				
# Subject	V1	V3	Δ(V3 - V1)	Δ(V3 -V1)%
1	0,330	0,175	-0,16	-46,97
2	0,220	0,088	-0,13	-60,00
3	0,375	0,240	-0,14	-36,00
4	0,246	0,091	-0,16	-63,01
5	0,390	0,206	-0,18	-47,18
6	0,330	0,327	0,00	-0,91
7	0,275	0,081	-0,19	-70,55
8	0,305	0,318	0,01	4,26
9	0,329	0,369	0,04	12,16
10	0,306	0,339	0,03	10,78
11	0,289	0,255	-0,03	-11,76
12	0,326	0,210	-0,12	-35,58
13	0,266	0,226	-0,04	-15,04
14	0,278	0,196	-0,08	-29,50
15	0,336	0,120	-0,22	-64,29
16	0,277	0,198	-0,08	-28,52
17	0,289	0,178	-0,11	-38,41
18	0,299	0,195	-0,10	-34,78
19	0,323	0,300	-0,02	-7,12
20	0,356	0,315	-0,04	-11,52
21	0,308	0,267	-0,04	-13,31
22	0,369	0,268	-0,10	-27,37
**Average**	0,310	0,226	-0,085	**-27,9**
**Std. Dev**	0,042	0,084	0,074	24,523
Nr of Subjects	22	22	22	
t-test	4,2307623			
degree of freedom	40			
p	0,0001319			

International Journal of *Molecular Sciences*

MDPI

Table S10. Effect of placebo on Uf 2-mm probe of treated volunteers at V1, V2 and V3. Uf 2-mm probe value for each volunteer and their variation are reported, along with average percentage variation value ± SD.

**Uf 2-mm probe V2 vs. V1 (baseline)** Placebo group				
**# Subject**	**V1**	**V2**	**Δ(V2 - V1)**	**Δ(V2 -V1)%**
1	0,277	0,242	-0,035	-12,6
2	0,281	0,296	0,015	5,3
3	0,260	0,236	-0,024	-9,2
4	0,229	0,199	-0,030	-13,1
5	0,302	0,319	0,017	5,6
6	0,260	0,256	-0,004	-1,5
7	0,281	0,276	-0,005	-1,8
8	0,269	0,258	-0,011	-4,1
9	0,257	0,225	-0,032	-12,5
10	0,254	0,226	-0,028	-11,0
11	0,276	0,256	-0,020	-7,2
12	0,235	0,234	-0,001	-0,4
13	0,284	0,269	-0,015	-5,3
14	0,325	0,347	0,022	6,8
15	0,211	0,198	-0,013	-6,2
16	0,325	0,365	0,040	12,3
17	0,254	0,221	-0,033	-13,0
18	0,347	0,356	0,009	2,6
19	0,244	0,288	0,044	18,0
20	0,263	0,253	-0,010	-3,8
21	0,230	0,232	0,002	0,9
**Average**	0,270	0,264	-0,005	**-2,4**
**Std. Dev**	0,034	0,049	0,023	8,623
Nr of Subjects	21	21	21	
t-test	0,4121325			
degree of freedom	38			
p	0,6825581			

**Uf 2-mm probe V3 vs. V1 (baseline)** Placebo group				
**# Subject**	**V1**	**V3**	**Δ(V3 - V1)**	**Δ(V3 -V1)%**
1	0,277	0,293	0,016	5,8
2	0,281	0,241	-0,040	-14,2
3	0,260	0,244	-0,016	-6,2
4	0,229	0,182	-0,047	-20,5
5	0,302	0,383	0,081	26,8
6	0,260	0,244	-0,016	-6,2
7	0,281	0,237	-0,044	-15,7
8	0,269	0,237	-0,032	-11,9
9	0,257	0,202	-0,055	-21,4
10	0,254	0,280	0,026	10,2
11	0,276	0,256	-0,020	-7,2
12	0,235	0,221	-0,014	-6,0
13	0,284	0,258	-0,026	-9,2
14	0,325	0,336	0,011	3,4
15	0,211	0,188	-0,023	-10,9
16	0,325	0,310	-0,015	-4,6
17	0,254	0,216	-0,038	-15,0
18	0,347	0,416	0,069	19,9
19	0,244	0,278	0,034	13,9
20	0,263	0,211	-0,052	-19,8
21	0,230	0,209	-0,021	-9,1
**Average**	0,270	0,259	-0,011	**-4,7**
**Std. Dev**	0,034	0,061	0,037	13,338
Nr of Subjects	21	21	21	
t-test	0,6931282			
degree of freedom	38			
p	0,492444			

Table S11. Anova test on 1.5% w/w PBA brown spots value vs placebo at V1, V2, and V3.

**Brown Spots average values at V1**		
	**PBA**	**Placebo**
	23,45	21,00
	22,80	21,70
	22,00	26,80
	15,90	17,80
	16,10	20,80
	23,90	21,70
	18,00	15,90
	18,00	11,80
	26,80	19,60
	21,30	21,30
	22,60	24,30
	24,10	24,40
	25,40	26,80
	26,70	23,40
	20,30	21,40
	20,60	19,00
	18,50	24,80
	19,50	19,10
	21,00	25,20
	20,00	21,03
	23,00	23,00
	24,10	
**Media gruppo**	21,547727	21,468095
**Media totale**	21,507911	
**Numero dati**	22	
**SSB**	0,06975387	
**SSx**	201,5823864	258,6995238
**SSW**	460,2819102	
**dfB**	1	
**dfS**	42	
**S^2B**	0,06975387	
**S^2W**	10,9590931	
**F**	0,006364931	
**Livello significatività**	0,05	
**pvalue**	0,936790753	
	NS	

**Brown Spots average percentage variation at V2**		
	**PBA**	**Placebo**
	-23,67	3,33
	-0,88	31,80
	-1,36	8,58
	-8,81	30,34
	-6,21	8,17
	-21,34	4,15
	-3,89	41,51
	-1,67	21,19
	-24,25	14,80
	-0,94	20,66
	-1,77	16,46
	-13,69	-6,97
	-8,27	8,21
	-10,49	-14,10
	-2,96	-1,87
	-6,80	-4,74
	0,00	-3,63
	-7,69	3,66
	-8,57	18,25
	-15,00	22,21
	-12,61	-4,35
	-21,99	-13,69
**Media gruppo**	-9,220226	10,364849
**Media totale**	0,572312	
**Numero dati**	22	
**SSB**	4219,32676	
**SSx**	1295,117918	4145,214665
**SSW**	5440,332582	
**dfB**	1	
**dfS**	42	
**S^2B**	4219,32676	
**S^2W**	129,5317282	
**F**	32,5736931	
**Livello significatività**	0,05	
**pvalue**	1,04613E-06	
	aaa	

**Brown Spots average percentage variation at V3**		
	**PBA**	**Placebo**
	-14,71	-2,38
	0,00	11,06
	-4,09	-16,42
	-10,06	8,99
	-24,84	2,40
	-11,72	8,76
	-21,11	3,14
	-10,56	28,81
	-32,09	-12,76
	-5,63	22,07
	-5,75	11,93
	-5,81	1,64
	-9,06	-0,75
	-4,49	10,68
	-1,48	2,80
	-16,02	28,95
	-23,24	-1,21
	-26,15	-2,62
	-2,86	10,71
	1,50	18,40
	2,17	0,00
	-13,69	
**Media gruppo**	-10,895343	6,391878
**Media totale**	-2,251732	
**Numero dati**	22	
**SSB**	3287,328031	
**SSx**	1950,690805	2759,87713
**SSW**	4710,567935	
**dfB**	1	
**dfS**	42	
**S^2B**	3287,328031	
**S^2W**	112,1563794	
**F**	29,3102189	
**Livello significatività**	0,05	
**pvalue**	2,75207E-06	
	aaa	

Table S12. Anova test on 1.5% w/w PBA individual typology angle values vs placebo at V1, V2, and V3.

Individual Typology Angle average values at V1		
	PBA	Placebo
	38	46
	49	39
	32	36
	41	32
	37	40
	44	32
	39	37
	39	35
	46	43
	51	50
	38	48
	43	49
	43	34
	48	39
	30	37
	31	44
	35	46
	41	42
	36	33
	41	34
	43	43
	44	
**Media gruppo**	40,409091	39,952381
**Media totale**	40,180736	
**Numero dati**	22	
**SSB**	2,29442383	
**SSx**	658,9181818	664,952381
**SSW**	1323,870563	
**dfB**	1	
**dfS**	42	
**S^2B**	2,29442383	
**S^2W**	31,52072769	
**F**	0,072790954	
**Livello significatività**	0,05	
**pvalue**	0,78863689	
	NS	

Individual Typology Angle average percentage variation at V2		
	PBA	Placebo
	11	-4
	18	-15
	22	-11
	2	31
	7	3
	15	-9
	8	8
	3	-11
	10	-7
	6	6
	13	-10
	10	0
	16	-12
	4	-3
	10	5
	10	-7
	11	0
	20	-5
	14	6
	2	-3
	2	-2
	5	
**Media gruppo**	9,934814	-1,947241
**Media totale**	3,993786	
**Numero dati**	22	
**SSB**	1553,015661	
**SSx**	721,390352	2053,813205
**SSW**	2775,203557	
**dfB**	1	
**dfS**	42	
**S^2B**	1553,015661	
**S^2W**	66,07627518	
**F**	23,50337783	
**Livello significatività**	0,05	
**pvalue**	1,74103E-05	
	aaa	

Individual Typology Angle average percentage variation at V3		
	PBA	Placebo
	5	2
	22	-10
	28	3
	27	3
	18	5
	9	-6
	3	0
	18	0
	5	-2
	8	0
	13	-6
	7	-2
	5	-9
	4	0
	10	3
	19	0
	20	-2
	7	0
	11	9
	5	-6
	23	0
	9	
**Media gruppo**	12,590455	-0,911062
**Media totale**	5,839697	
**Numero dati**	22	
**SSB**	2005,20069	
**SSx**	1325,234701	429,800948
**SSW**	1755,035649	
**dfB**	1	
**dfS**	42	
**S^2B**	2005,20069	
**S^2W**	41,78656307	
**F**	47,98673407	
**Livello significatività**	0,05	
**pvalue**	1,84706E-08	
	aaa	

Table S13. Anova test on 1.5% w/w PBA UV spots values vs placebo at V1, V2, and V3.

UV Spots average values at V1		
	PBA	Placebo
	25,4	21,3
	22,8	25,7
	28,9	26,1
	27,3	21,1
	22,5	27,2
	20,7	28,2
	22,8	20,7
	27,0	26,5
	25,6	23,3
	24,4	29,3
	23,9	22,4
	24,2	22,1
	22,5	23,5
	25,0	26,7
	27,6	22,6
	26,2	26,2
	22,2	28,3
	23,8	25,3
	23,5	21,2
	22,1	28,2
	22,8	20,5
	25,0	
**Media gruppo**	24,372727	24,590476
**Media totale**	24,481602	
**Numero dati**	22	
**SSB**	0,521560503	
**SSx**	92,22363636	167,6980952
**SSW**	259,9217316	
**dfB**	1	
**dfS**	42	
**S^2B**	0,521560503	
**S^2W**	6,188612657	
**F**	0,084277451	
**Livello significatività**	0,05	
**pvalue**	0,773010907	
	NS	

UV Spots average percentage variation at V2		
	PBA	Placebo
	-13,4	8,5
	-10,5	1,2
	-18,3	-3,4
	-17,6	10,0
	-7,2	1,1
	-3,9	-6,7
	0,8	18,8
	-12,6	-4,9
	-15,6	16,3
	-11,9	-8,2
	-2,3	1,3
	2,4	5,9
	4,4	-0,9
	-12,0	10,5
	-14,5	-2,7
	-4,2	-3,1
	1,8	0,0
	4,6	13,0
	-13,2	16,5
	-7,2	4,3
	-3,5	-1,0
	-6,8	
**Media gruppo**	-7,302002	3,643919
**Media totale**	-1,829042	
**Numero dati**	22	
**SSB**	1317,944967	
**SSx**	1085,956801	1288,824006
**SSW**	2374,780807	
**dfB**	1	
**dfS**	42	
**S^2B**	1317,944967	
**S^2W**	56,54240017	
**F**	23,30896748	
**Livello significatività**	0,05	
**pvalue**	1,85758E-05	
	aaa	

UV Spots average percentage variation at V3		
	PBA	Placebo
	-9,1	3,8
	0,0	-1,2
	-18,3	2,7
	-14,7	3,8
	-8,6	4,0
	-5,7	-7,1
	0,8	13,5
	-4,6	-3,8
	-3,1	-6,4
	-8,4	0,7
	-8,9	1,3
	-5,6	5,0
	-6,6	3,0
	-7,3	-2,6
	-6,9	2,7
	-11,9	4,2
	7,6	-2,1
	-8,0	4,0
	-11,5	1,9
	-8,1	-0,4
	-5,7	8,3
	-3,6	
**Media gruppo**	-6,737040	1,676002
**Media totale**	-2,530519	
**Numero dati**	22	
**SSB**	778,5721073	
**SSx**	606,3167447	443,3836683
**SSW**	1049,700413	
**dfB**	1	
**dfS**	42	
**S^2B**	778,5721073	
**S^2W**	24,99286698	
**F**	31,15177254	
**Livello significatività**	0,05	
**pvalue**	1,58548E-06	
	aaa	

International Journal of *Molecular Sciences*

MDPI

Table S14. Anova test on 1.5% w/w PBA Ua/Uf 2-mm probe values vs placebo at V1, V2, and V3.

Ua/Uf 2-mm probe average values at V1		
	PBA	Placebo
	0,556	0,561
	0,421	0,438
	0,473	0,535
	0,597	0,587
	0,554	0,404
	0,550	0,541
	0,500	0,578
	0,538	0,491
	0,448	0,531
	0,505	0,448
	0,402	0,551
	0,496	0,585
	0,561	0,574
	0,523	0,489
	0,544	0,425
	0,552	0,566
	0,436	0,455
	0,566	0,411
	0,459	0,475
	0,448	0,513
	0,521	0,435
	0,578	
**Media gruppo**	0,510364	0,504429
**Media totale**	0,507396	
**Numero dati**	22	
**SSB**	0,000387475	
**SSx**	0,064613091	0,077043143
**SSW**	0,141656234	
**dfB**	1	
**dfS**	42	
**S^2B**	0,000387475	
**S^2W**	0,003372767	
**F**	0,114883388	
**Livello significatività**	0,05	
**pvalue**	0,73634037	
	NS	

Ua/Uf 2-mm probe average percentage variation at V2		
	PBA	Placebo
	7,374	1,604
	12,589	-1,370
	-3,594	5,421
	48,576	-7,666
	-1,805	7,921
	0,545	0,555
	8,200	1,038
	5,204	10,183
	68,527	6,591
	25,149	2,009
	13,184	13,430
	5,040	-10,940
	28,520	-5,052
	12,428	9,407
	5,699	6,588
	21,014	-6,714
	17,431	-4,176
	1,943	14,112
	17,429	10,737
	18,080	10,721
	26,679	2,069
	15,744	
**Media gruppo**	16,089059	3,165147
**Media totale**	9,627103	
**Numero dati**	22	
**SSB**	1837,302755	
**SSx**	5855,959761	1051,166603
**SSW**	6907,126364	
**dfB**	1	
**dfS**	42	
**S^2B**	1837,302755	
**S^2W**	164,4553896	
**F**	11,17204343	
**Livello significatività**	0,05	
**pvalue**	0,001753351	
	aa	

Ua/Uf 2-mm probe average percentage variation atV3		
	PBA	Placebo
	-19,604	-6,061
	43,468	28,311
	-12,896	25,981
	16,415	-10,051
	46,209	7,921
	16,909	-16,081
	26,200	3,114
	6,134	20,570
	-1,786	0,942
	0,792	7,589
	77,114	19,964
	38,306	-22,222
	40,107	-1,916
	69,981	-6,748
	22,243	-32,000
	60,688	37,633
	61,009	25,055
	22,085	-2,433
	57,298	8,842
	91,071	1,949
	69,482	-18,621
	49,481	
**Media gruppo**	35,486712	3,416031
**Media totale**	19,451372	
**Numero dati**	22	
**SSB**	11313,81474	
**SSx**	19314,71809	6626,059555
**SSW**	25940,77764	
**dfB**	1	
**dfS**	42	
**S^2B**	11313,81474	
**S^2W**	617,6375629	
**F**	18,31788644	
**Livello significatività**	0,05	
**pvalue**	0,000105937	
	aaa	

Table S15. Anova test on 1.5% w/w PBA Uf 2-mm probe value vs placebo at V1, V2, and V3.

Uf 2-mm probe average values at V1		
	PBA	Placebo
	0,330	0,277
	0,220	0,281
	0,375	0,260
	0,246	0,229
	0,390	0,302
	0,330	0,260
	0,275	0,281
	0,305	0,269
	0,329	0,257
	0,306	0,254
	0,289	0,276
	0,326	0,235
	0,266	0,284
	0,278	0,325
	0,336	0,211
	0,277	0,325
	0,289	0,254
	0,299	0,347
	0,323	0,244
	0,356	0,263
	0,308	0,230
	0,369	
**Media gruppo**	0,310091	0,269714
**Media totale**	0,289903	
**Numero dati**	22	
**SSB**	0,017932989	
**SSx**	0,037001818	0,023118286
**SSW**	0,060120104	
**dfB**	1	
**dfS**	42	
**S^2B**	0,017932989	
**S^2W**	0,001431431	
**F**	12,52801449	
**Livello significatività**	0,05	
**pvalue**	0,000994924	
	aaa	

Uf 2-mm probe average percentage variation at V2		
	PBA	Placebo
	-15,152	-12,635
	14,545	5,338
	-1,867	-9,231
	-35,772	-13,100
	-26,923	5,629
	-6,667	-1,538
	0,364	-1,779
	-6,230	-4,089
	14,894	-12,451
	-40,850	-11,024
	-12,111	-7,246
	-12,577	-0,426
	-10,150	-5,282
	-8,633	6,769
	-19,940	-6,161
	-9,386	12,308
	-8,651	-12,992
	-16,388	2,594
	-32,508	18,033
	-9,831	-3,802
	-2,922	0,870
	-10,027	
**Media gruppo**	-11,671875	-2,391314
**Media totale**	-7,031595	
**Numero dati**	22	
**SSB**	947,4169246	
**SSx**	3999,933882	1487,154915
**SSW**	5487,088797	
**dfB**	1	
**dfS**	42	
**S^2B**	947,4169246	
**S^2W**	130,6449713	
**F**	7,251843794	
**Livello significatività**	0,05	
**pvalue**	0,010132005	
	aa	

Uf 2-mm probe average percentage variation at V3		
	PBA	Placebo
	-46,970	5,776
	-60,000	-14,235
	-36,000	7,965
	-63,008	-20,524
	-47,179	26,821
	-0,909	-6,154
	-70,545	-15,658
	4,262	-11,896
	12,158	-21,401
	10,784	10,236
	-11,765	-7,246
	-35,583	-5,957
	-15,038	-9,155
	-29,496	3,385
	-64,286	-10,900
	-28,520	-4,615
	-38,408	-14,961
	-34,783	19,885
	-7,121	13,934
	-11,517	-19,772
	-13,312	-9,130
	-27,371	
**Media gruppo**	-27,936626	-3,981113
**Media totale**	-15,958869	
**Numero dati**	22	
**SSB**	6312,532365	
**SSx**	12629,35036	3705,622289
**SSW**	16334,97265	
**dfB**	1	
**dfS**	42	
**S^2B**	6312,532365	
**S^2W**	388,9279202	
**F**	16,23059708	
**Livello significatività**	0,05	
**pvalue**	0,000230419	
	aaa	
